# Investigating Length of Stay Patterns and Its Predictors in the South Wales Trauma Network

**DOI:** 10.1177/27536351241237866

**Published:** 2024-03-19

**Authors:** Zihao Wang, Bahman Rostami-Tabar, Jane Haider, Mohamed Naim, Javvad Haider

**Affiliations:** 1 Cardiff Business School, Cardiff University, Cardiff, UK; 2 National Rehabilitation Centre, Nottingham University Hospitals NHS Trust, Nottingham, UK

**Keywords:** Trauma, length of stay, sehabilitation

## Abstract

**Background:**

Length of stay (LOS) is frequently employed as a performance metric for trauma care. Following the establishment of the trauma network worldwide, the assessment and prediction of LOS in different levels of trauma centres have been extensively studied. However, assessing the total patient length of stay from a whole trauma network perspective is unclear. The objective of this study was to systematically analyse the overall Length of Stay (LOS) pattern within the SWTN before its establishment and in the immediate time after its foundation and, secondly, to assess the association between relevant impact factors and LOS.

**Methodology:**

A retrospective secondary analysis based on the trauma admission dataset from Trauma Audit and Research Network(TARN) dataset was conducted. The studied sample covered around 18000 patients admitted to trauma centres from South Wales Major trauma network between January 2012 and October 2021. The primary outcome is the total length of stay in the trauma network. Statistical tests were applied to examine the difference between normal and outlier LOS. Data visualisation was utilised to demonstrate the LOS patterns and potential association between LOS and relevant demographic and clinical predictors.

**Results:**

The distribution of length of stay in SWTN follows a right-skewed distribution with a median of 10 (IQR, 5–18) and a mean of 15.92 days. There were 1520 patients with outliers for LOS. A significant difference (p¡ 0.05) was found between the normal and outlier groups of LOS based on demographic (age, gender and residential information) and clinical characteristics(ward type, maximum of anatomically-based injury severity score(AIS) and probability of survival). Age group, maximum AIS score on specific injured region, ward type and its interaction effect with the number of admissions may associated with the LOS. Specifically, patients admitted to the geriatric ward exhibited notably prolonged LOS, and individuals with more than 2 admissions to long-term care and recovery-related wards such as neurosurgical rehabilitation, spinal injuries and burns wards also displayed elevated LOS.

**Conclusion:**

Our finding supports prior evidence indicating elderly people are vulnerable to longer stays. Moreover, concerning the types of admission wards, patients admitted to rehabilitation wards who underwent more than 2 hospitalisations also faced an increased risk of prolonged stay. Based on these results, policymakers and healthcare providers should contemplate expanding the allocation of medical resources to this demographic to mitigate the length of stay and optimise associated healthcare costs.

## Introduction

Trauma is known to be one of the leading causes of mortality and disability.^1^ It is now well established that outcomes following trauma can be improved by reorganisation of trauma systems. Specifically, optimal outcomes globally have been demonstrated when trauma care within a region is provided by a network of hospitals with a major trauma centre (MTC) as a central hub.^2-4^ In England, there has been an improvement in mortality and disability related to traumatic injuries with a reduction in rank and burden from road injuries in particular.^5^ This can be attributed to the introduction of regional trauma networks and major trauma centres in 2012 following the NCEPOD 2007 report, which identified avoidable deaths occurring due to the majority of major trauma patients receiving a suboptimal standard of care. A landmark study in 2018 estimated that there were an additional 1600 patients surviving major trauma injuries in England since major trauma centres were established in 2012.^6^ The South Wales Trauma Network (SWTN) was established in September 2020 with a major trauma centre (MTC) located in the University Hospital of Wales in Cardiff.

Length of stay (LOS) is a frequently used metric to judge the performance of trauma care. Studies show contradictory results regarding the effect of prolonged LOS on mortality. Some studies in hospitalised trauma patients show longer LOS to be an independent predictor of higher hospital mortality.^7^ while others report improved survival outcomes with prolonged LOS.^8^ A reduction in LOS is however certainly accompanied by a significant reduction in healthcare costs.^9^

Previous studies have identified various factors influencing LOS. Injury severity, surgical operations, complications and type of injury have frequently been cited as predictors of LOS.^10-12^ These studies, however, often look at LOS in a particular unit, for example, emergency department or intensive care unit, rather than overall hospital LOS. Also, certain factors related to the trauma patient journey have not been studied extensively, for example, type and timing of specialist ward environment post-emergency/intensive care unit

The objective of this study is firstly to describe the patient length of stay under the impact of demographical and clinical characteristics of trauma patients and secondly to identify potential predictors that contribute to the length of stay among patients admitted to hospitals in South Wales covering around 18000 admissions during the period January 2012 to October 2021. This study establishes the initial basis for future investigations, focussing specifically on long-term periods after the launch of the SWTN and MTC in Cardiff.

## Materials and Methods

### Data source

The Trauma Audit and Research Network (TARN) is the National Clinical Audit for Trauma Care that gathers data on the acute treatment pathway of patients suffering from major trauma injury who have more than 3 overnight stays or are admitted to intensive care (including transferred patients). Each Trauma Unit or Major Trauma Centre that is part of a Major Trauma Network is required to provide data. TARN gathers information from 218 hospitals in England, Wales, Northern Ireland and Ireland. This is used to monitor trauma treatment and enhance trauma services.

We obtained access to the TARN dataset for Major Trauma admissions in South Wales through the Secure Anonymised Information Linkage (SAIL) databank. SAIL databank is a de-identified and linkable dataset of patient records in Wales. SAIL makes selected datasets available to researchers in anonymised form with extensive safeguards in place. SAIL is hosted by Swansea University and receives funding from Health and Care Research Wales and UK Research and Innovation’s (UKRI) Economic and Social Research Council (ESRC). We are accredited with the Office for National Statistics (ONS) and submit an application to the SAIL data bank. A data-sharing agreement was set up between TARN and SAIL supported by a Confidentiality Advisory Group committee approval from the Health Research Authority in the UK permitting access to this dataset for the purposes of this research project run through Cardiff University. The data used in this paper included around 18 000 admissions to certain institutions within the South Wales Major Trauma Network between January 2012 and October 2021. These institutions encompass a Major Trauma Centre, Specialised acute hospitals with various trauma units, a rural trauma facility and a local emergency hospital.

### Data pre-processing

**Figure 1.**
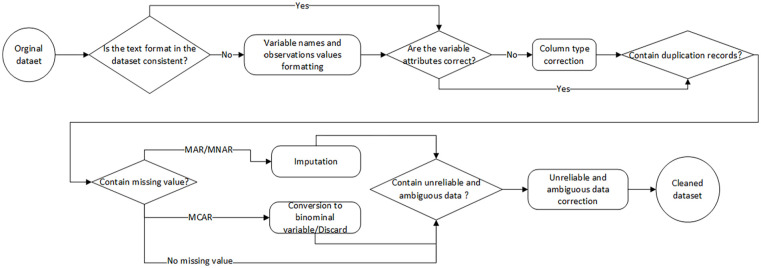
Data preparation flow diagram showing data pre-processing steps.

We examined the data quality of the obtained raw data and performed data cleaning. After imputing or removing missing values and correcting implausible data (Figure 1), the final dataset contains 71 variables and 17 966 observations.

### Variables

**Figure 2.**
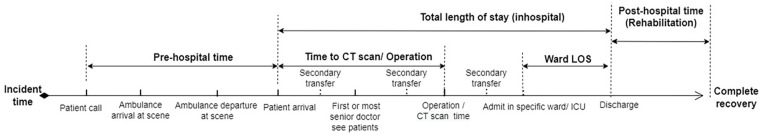
Trauma pathway illustration.

The response variable is the total length of stay (LOS) in the trauma network, which is defined as the duration from a patient’s arrival at a specific healthcare facility until their discharge, encompassing a series of clinical events and multiple transfers or admissions in different hospitals (Figure 2). Predictors of interest included patient demographics, vital sign measurement, injury severity score, maximum AIS score based on different injured regions, most severely injured regions and ward types based on the first 3 admissions records. A list of summary statistics is presented as the median and interquartile range (IQR) values for continuous variables and counts (percentages) for categorical variables.

### Data analysis

Data was analysed and visualised using R 4.2.0 and Rstudio. A histogram was employed to visualise the distribution of LOS, accompanied by a statistical summary for an in-depth understanding. Outliers in the LOS data were precisely defined for accurate differentiation within the dataset. Regarding the identified outliers, a systematic comparison was conducted to examine the demographic and clinical characteristics distinguishing the outlier group from the main distribution. The median and interquartile range (IQR) were calculated for numerical variables, while percentages were used for categorical variables. Further statistical analysis was carried out to examine the difference between these 2 groups. Chi-Square and Kruskal-Wallis tests of independence were applied to categorical variables, while a Mann-Whitney *U* test was employed for continuous variables, with a significance threshold of *P* < .05.

Due to the right-skewed distribution of LOS, a natural logarithm transformation was utilised in the multivariate visualisation, serving to enhance visual clarity and promote data comparability while describing the LOS. Several box plots and density graphs were utilised to capture potential associations between LOS and key variables of interest. The log transformed-LOS based on age group, times of operation performed, AIS severity score and ward type were demonstrated separately.

## Results

This section presents the results of a statistical analysis and data visualisation focussing on examining baseline patient characteristics and their relationship with length of Stay (LOS).

### General characteristics of trauma patients

Out of the 17 966 patients admitted between January 2012 and October 2021, the majority, accounting for 93.37%, were Welsh residents, whereas the remaining 6.63% were non-residents. In addition, 79% of reported trauma incidents occurred in Wales, while 21% did not.

Table 1 presents an overview of the variables available in the dataset, along with their corresponding descriptive statistics. Regarding gender, a significant difference emerged between the 2 LOS groups(*P* < .0001). Specifically, the female ratio in the outlier LOS group was 53.68% compared to 47.1% in the normal LOS group, with female patients exhibiting longer LOS, whereas the converse distribution was observed in the normal LOS group. Besides that, there was also a significant difference in age groups between the 2 LOS groups (*P* < .0001). Patients over 75 years old accounted for the majority (59.14%) of the outlier LOS groups, followed by patients in the age group of 65 to 74 years (15%). Additionally, there was a disparity in the distribution of discharge status between the 2 groups. In the normal LOS group, 9.2% of the patients did not survive to discharge, whereas all patients in the outlier LOS group had a favourable outcome.

**Table 1.** Demographic characteristics comparison within the 2 los groups (*P*-value is based on the Chi-square test).

	Length of stay ⩽37 days	Length of stay >37 days	*P*
Gender			<.0001
Male	8700 (52.9%)	704 (46.32%)	
Female	7745 (47.1%)	816 (53.68%)	
Age group (years)			<.0001
1-44	3072 (20.93%)	141 (9.67%)	
45-54	1835 (11.16%)	88 (5.79%)	
55-64	2534 (15.41%)	158 (10.39%)	
65-74	2202 (13.39%)	228 (15.00%)	
75 and over	6432 (39.11%)	899 (59.14%)	
Status of discharge			<.0001
Alive	15265 (92.82%)	1520 (100%)	
Dead	1180 (7.18%)		

A breakdown of descriptive statistics of other demographic variables is provided in the Supplemental Section (Supplemental Table 4).

### Length of stay

**Figure 3.**
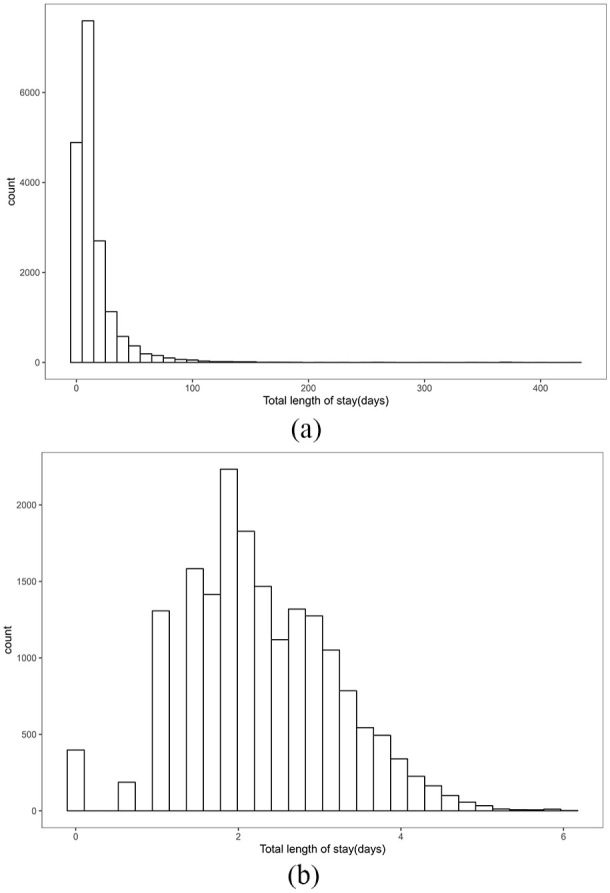
Distribution of the length of stay. (a) Original (b) log-transformed.

The total length of stay exhibits a rightward skewed distribution (Figure 3) with a median of 10 (IQR, 5–18) and a mean of 15.92 ± 21.65 days. The outlier of the length of stay is defined as cases exceeding 1.5 times the IQR range, which was calculated to be 37 days (around the 91th percentile for patients). Around 1520 patients(8.5%) were identified in the outlier LOS group with a stay longer than 37 days vs 16445 patients (91.5%) whose stay in the trauma network was shorter than 37 days.

Patients within the outlier longer LOS group had marked differences in baseline characteristics compared with those in the shorter stay (Tables 1-3). It needs to be pointed out that, however, minor differences in patient characteristics between patients with and without length of stay outliers can be statistically significant due to the large sample size of the population studied.

**Table 2.** Injury severity comparison with or without los outliers (*p*-value is based on the Mann-Whitney *U* test and Chi-square test).

	Length of stay ⩽37 days	Length of stay >37 days	*P*
ISS (median ± iqr)	9 [9-17]	10 [9-20]	<.0001
Pulse (median ± iqr)	82 [71-94]	83 [71-96]	.1205
Respiratory rate (median ± iqr)	18 [16-20]	18 [17-20]	.0030
SBP	137 [121-156]	140 [120-159]	.0639
GCS (n%)			.0408
3-8	424 (2.58%)	54 (3.55%)	
9-12	412 (2.51%)	<45 (2.96%)	
13-15	15609 (94.92%)	<1421 (93.49%)	
Most severe injured region (n%)			<.0001
Abdomen	377 (2.32%)	11 (0.73%)	
Chest	2995 (18.46%)	142 (9.39%)	
Head	3352 (20.66%)	329 (21.76%)	
Limbs	5795 (35.72%)	603 (39.88%)	
Multiple	1490 (9.19%)	171 (11.31%)	
Spine	2213 (13.64%)	256 (16.93%)	
AIS maximum severity in head (n%)			.0031
1-2	892 (20.00%)	112 (22.76%)	
3-4	2352 (52.74%)	220 (44.72%)	
5-6	1216 (27.26%)	160 (32.52%)	
AIS maximum severity in thorax (n%)			.0013
1-2	1076 (23.96%)	96 (27.20%)	
3-4	3327 (74.10%)	241 (68.27%)	
5-6	87 (1.94%)	16 (4.53%)	
AIS maximum severity in abdomen (n%)			.2188
1-2	505 (49.95%)	32 (38.55%)	
3-4	477 (47.18%)	51 (61.45%)	
5-6	29 (2.87%)		
AIS maximum severity in spine (n%)			<.0001
1-2	2628 (66.82%)	240 (50.21%)	
3-4	1273 (32.37%)	224 (46.86%)	
5-6	74 (0.81%)	14 (2.93%)	
AIS maximum severity in pelvis (n%)			.001
1-2	1735 (80.74%)	233 (76.64%)	
3-4	340 (15.82%)	47 (15.46%)	
5-6	74 (3.44%)	24 (7.89%)	
AIS maximum severity in limbs (n%)			.0013
1-2	3555 (43.24%)	40 (37.43%)	
3-4	4667 (56.76%)	56 (62.57%)	

**Table 3.** Comparison of ward types based on all admissions records(*P*-value is based on the Chi-square test).

	Length of stay ⩽37 days	Length of stay >37 days	*P*
Ward type			<.00001
Cardiothoracic	515 (3.22%)	22 (1.38%)	
Emergency admissions unit (EAU)	1005 (6.29%)	65 (4.07%)	
General acute (inc. paediatric)	677 (4.24%)	67 (4.20%)	
General paediatric	751 (0.45%)	69 (0.07%)	
Geriatric	297 (1.86%)	125 (7.83%)	
Major trauma ward	365 (2.28%)	10 (0.63%)	
Medical ward (inc. palliative care)	1379 (8.63%)	234 (14.65%)	
Neurosurgical rehabilitation ward	234 (1.46%)	19 (1.19%)	
Orthopaedic (inc. paediatric)	8319 (52.04%)	788 (50.72%)	
Plastic surgery	127 (0.79%)	11 (0.69%)	
Spinal injuries unit	337 (1.85%)	80 (4.41%)	
Surgical ward (inc. paediatric)	1761 (11.02%)	71 (4.45%)	
Surgical ward (inc. paediatric)	218 (1.36%)	14 (0.88%)	

#### Clinical and injury severity characteristics

Table 2 shows the statistical significance of the impact of injury severity on LOS ≤37 and >37 .

Aside from GCS (*P* = .04) and Respiratory Rate (*P* = .003), no significant differences were observed in the physiological indicators between the 2 LOS groups. Specifically, for patients with GCS scores in the range of 3 to 8, the outlier los groups exhibited a proportion of 2.51%, while the other had a proportion of 3.55%. For patients with GCS scores between 13 and 15, a statistically significant difference was also observed, with proportions of 94.92% for normal LOS and 93.49% for outlier LOS.

The most severely injured body region differed significantly between the 2 groups (*P* < .0001). Among these, the outlier LOS group exhibited a significantly higher proportion of limbs (35.72%), multiple (9.19%) and other (16.84%) injuries compared to the regular LOS group, whereas the opposite trend was observed for injuries in the chest area (9.34%). The level of the first doctor to see the patients in ED of the admitted patients did not differ significantly between the groups.

Regarding injury severity, the ISS score for the outlier LOS group was relatively high, measuring 10 [9–20]. In addition, besides injuries in the abdominal region, a notable discrepancy in the maximum severity of AIS was evident across all other injury regions when comparing the 2 groups (*P* < .05). Specifically, the proportion of patients within the outlier LOS group surpassed that of the regular LOS group for each AIS score falling within the range of 5 to 6. This observation underscores the significance of the association between maximum AIS severity in the specific injured region and the patient’s length of stay.

#### Characteristics of ward types for the admissions

Owing to data confidentiality regulations on SAIL platforms, specific subgroups(Burns, Coronary Care Unit,Post Anaesthetic Care Unit and Maxillofacial) with values below 10 were merged into ’Other’ categories.

According to the Table 3, a statistically significant difference(*P* < .0001) was observed in the length of stay between the 2 groups based on the type of ward where the patients were admitted for the initial 3 occurrences. Half of the patients(52.04% and 50.72%) in both LOS groups had been admitted to the orthopaedic ward. A notably higher proportion of patients with a length of stay (LOS) exceeding 37 days had been admitted to the Medical Ward (14.65%), Geriatric ward(7.83%)and the Spinal Injuries Unit (5%) compared to the shorter LOS groups. In contrast, for the other types of wards, the outlier LOS group had a significantly lower proportion of admissions to Cardiothoracic Wards (1.38%), Major Trauma Wards (0.63%), Emergency admissions (4.07%) and surgical ward(4.45%) than the lower LOS group.

### Age group

**Figure 4.**
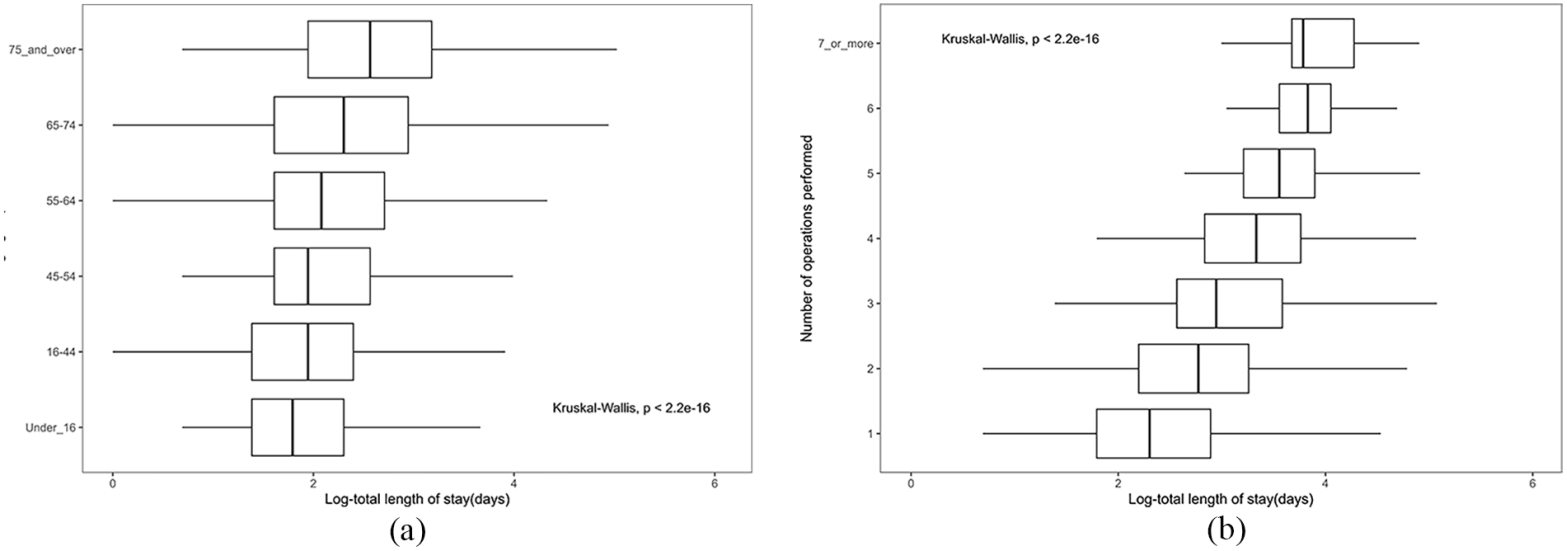
Visualisation of LOS based on demographic and admission characteristics.(The *x* axis represents the length of stay, while the y axis refers to the category based on age groups and times of operation performed.) (a) Distribution of LOS based on age group. (b) Distribution of LOS based on times of operation performed.

Figure 4a depicts the distribution of the LOS across 6 distinct age groups. As age increases, the median and IQR of LOS show substantial growth. Specifically, the age group ‘75 and over’ exhibits the highest median and IQR(13[7-24]) of LOS. The p-value (*P* < 2.2e-16) from the Kruskal-Wallis test showed that the variations in LOS between age groups were statistically significant, which indicates that age is a factor with a considerable impact on the LOS.

### Total operation performed in total length of stay

Figure 4b illustrates the distribution of the total length of stay among different numbers of operations performed: The x-axis represents how many operations were performed, while the y-axis represents the LOS. It can be seen that most patients underwent either 1 (5763 cases) or 2 (715 cases) surgical operations. Interestingly, this group exhibited markedly lower median LOS and narrower upper and lower quartiles than the other cohorts. It’s noteworthy, however, that there were a significant number of unusually high outliers within these categories. In contrast, with an increasing number of surgeries (ranging from 3 to 6), there was a progressive rise in both the median LOS and IQRs. Furthermore, the Kruskal-Wallis test (*P* < .05) underscored a significant difference in the distribution of LOS among individuals who had experienced increasing number of surgical operations.

### Ward type

**Figure 5.**
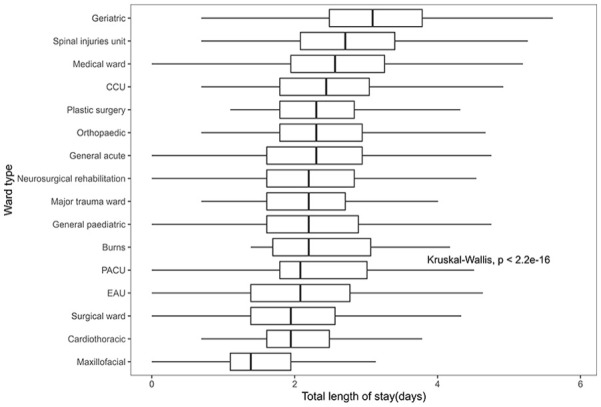
Distribution of LOS based on the total ward type.

Figure 5 presents the distribution of length of stay (LOS) based on the type of ward to which patients were admitted. When arranging the wards in descending order of median LOS, it becomes evident that patients admitted to geriatric wards experienced higher median LOS values, along with wider IQRs, compared to the other groups. Subsequently, the median length of stay was also notably higher for patients admitted to the spinal injury unit, medical ward and CCU (Coronary Care Unit) compared to the remaining groups. Plastic surgery, orthopaedic and general acute wards followed closely, which exhibited similar median LOS values. Notably, the orthopaedic wards accounted for the largest sample size, with 9129 admissions to this sub-type of the ward and also had a notable number of outliers in terms of hospital stay duration. Continuing down the order were neurosurgical rehabilitation wards, major trauma units, general paediatrics and burns wards, which displayed relatively similar median LOS values but with variations in distribution. The burns ward’s distribution, in particular, leaned towards a right-skewed pattern. Subsequent groups with lower median LOS included PACU (Post-Anesthesia Care Unit), EAU (Emergency Assessment Unit), surgical ward and cardiac ward and they also exhibited some higher outliers. The maxillofacial ward recorded the lowest median LOS, accompanied by relatively narrower IQRs.

**Figure 6.**
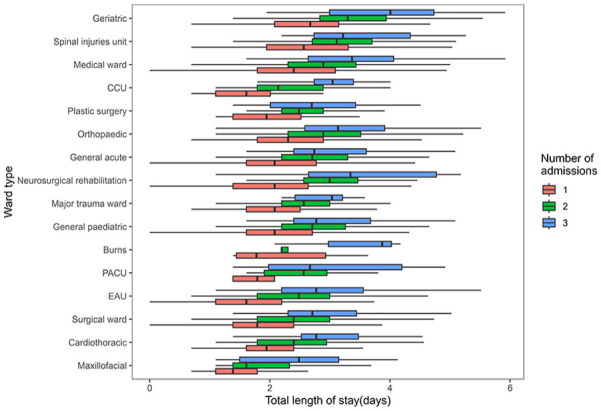
Distribution of LOS based on the total ward type and admission times.

Recognising that an elevated number of patient ward transfers is associated with longer stays, we have incorporated in Figure 6 a visualisation of how the Length of Stay (LOS) is affected by both the total number of patient admissions and the specific type of ward to which they were admitted. This analysis effectively captures the interaction effect of these factors on the LOS.As shown in Figure 6, regardless of the number of hospital admissions, patients admitted to the geriatric ward consistently exhibited a higher median length of stay than patients admitted to other types of wards on each ward transfer admission occasion. Nevertheless, certain patients admitted to wards specialising in long-term treatment and rehabilitation, such as neurosurgical rehabilitation, spinal injury unit and burns ward and who had more than 2 ward transfers experienced a longer stay in comparison to patients in other wards over the same period. Specifically, excluding the geriatric ward, patients admitted to the neurosurgical and rehabilitation ward with 2 or 3 hospital admissions had the second-highest median length of stay compared to patients in other wards. Among them, individuals with 3 hospital admissions exhibited a greater right-skewed distribution of hospitalisation days and a broader IQR compared to those in other groups.

### Injury severity based on body region

#### Maximum AIS score based on different injured regions

**Figure 7.**
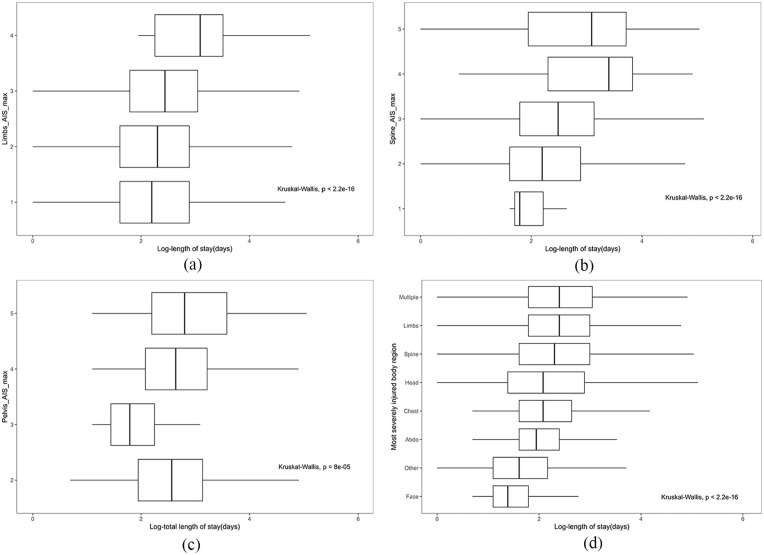
visualisation of LOS based on injury severity and injured body region. (*Y*-axis indicates the maximum AIS severity of a specific injured region or the most severe injured region, while *x*-axis indicates the length of stay). (a) Distribution of LOS in the maximum AIS of limbs. (b) Distribution of LOS in the maximum AIS of spine. (c) Distribution of LOS in the maximum AIS of pelvis. (d) Distribution of LOS in the most severed injured body region.

Figure 7a-c demonstrate the LOS variation based on the maximum AIS severity across limbs, spine and pelvis. As shown in Figure 7a, the patient’s maximum AIS severity of limbs ranged from 1 to 4, and there was a noticeable upward trend in the median length of hospital stay as the maximum severity level increased. Among the 9062 patients with varying degrees of limb injury severity, fewer cases(less than 10) exhibited an AIS of 4, whereas the majority (5186) registered an AIS of 3. According to the Figure 7b, among the cohort of 4408 patients presenting with spinal injuries, the range of AIS scores spanned from 1 to 6. The majority held a maximum AIS severity of 2 or 3, comprising 2865 and 1376 cases, respectively, and they exhibited a lower median length of stay compared to patients with AIS scores of 4 and 5. As depicted in Figure 7c, out of the total cohort, 2453 individuals presented varying degrees of pelvic impairment, resulting in AIS scores spanning from 2 to 5. Notably, within the group characterised by an AIS of 3, the median length of stay and the range of variation were significantly lower than those observed in the remaining AIS groups. Conversely, most individuals (1968 cases) with an AIS of 2 demonstrated a lower median length of stay than those with AIS scores of 4 and 5. In particular, ‘98 individuals with an AIS of 5 exhibited the highest median length of stay within this cohort’. The Kruskal-Wallis test yielded a significant result (p ¡ 0.05), indicating a statistically significant difference in the distribution of length of stay among the maximum severity of AIS groups

#### Most severe AIS score on corresponding injury region

Figure 7d illustrates the distribution of length of stay across various regions with the most severe injuries. Notably, the median number of days spent in the trauma network was highest when the region of most severe injury was ’multiple’ (1661 cases). Following closely, the ’limb’ region (6399 cases) exhibited a similar median duration of stay, albeit with a slightly narrower interquartile range (IQR). Subsequent median rankings, in descending order, were observed for ‘spine’ (2469 cases), ‘head’ (3681 cases), ‘chest’ (3137 cases), ‘abdomen’ (388 cases) and ‘other’ regions (66 cases). Conversely, the lowest median duration, accompanied by the narrowest range of variation, was found when the most severely injured region was ’face’ (168 cases). It is noteworthy that each group displayed several notable high outliers. Similarly, the Kruskal-Wallis analysis gave a significant result (*P* < .05), which means a statistically significant difference in the length of stay among the groups with the most severe injury.

## Discussion

This study aimed to investigate the factors influencing length of stay (LOS) in the context of the South Wales Trauma Network (SWTN), primarily using data from before its establishment and including early observations post-establishment. By employing retrospective data, our initial data visualisation analysis revealed compelling trends. Specifically, the visualisation results strongly suggest a potential association between LOS and key factors, including age group, the maximum AIS severity in different injured regions, the number of surgical operations performed, the type of ward admitted to and the number of ward transfers.

Age is the feature that appears most frequently in LOS analysis.^13^ Our study found a significant difference in LOS among different age groups, which aligns with previous studies.^12,14,15^. In our cohort, increasing age was associated with longer LOS. This finding may be attributed to the elderly population’s potentially multiple comorbid conditions and increasing social support requirements.^16,17^ In addition, a study conducted by Tal^18^ identified additional risk factors for prolonged LOS in older individuals, including the consumption of a high number of drugs ( ⩾ 5 drugs), non-independent functional status and specific admission diagnoses such as urinary tract infection (UTI), pneumonia and malignancy. Therefore, it is crucial to provide effective care and rehabilitation services to elderly trauma patients to reduce the duration of their hospital stay.

The visual analysis of the relationship between ward type and LOS suggests that patients admitted to certain wards(eg, geriatric, spinal injury unit and medical ward) may be more prone to experiencing prolonged LOS. Universally we observed that LOS significantly increased with the number of ward admissions within the same overall patient stay. However, it is noteworthy that the most dramatic increase in LOS was in the group of patients with 2 or 3 documented ward admissions leading up to admission to the neurosurgical ward. Given the complexity of neurological rehabilitation and the varying degrees of injury severity among patients admitted to a neurosurgical rehabilitation ward, the recovery journey for each individual is vastly different.

In our study, we observed that 90% of patients were discharged following their initial admission to a neurosurgical rehabilitation ward. This may reflect a group of patients with more straightforward needs with lesser neurological impairment. For patients with 2 or 3 ward transfer admissions who are subsequently admitted to neurosurgical rehabilitation, at least 1 of their ward admissions required acute inpatient treatment on another type of ward. There are several explanations for the dramatic rise in LOS in these patients. The initial stay in another high dependency ward (ie, ICU) may reflect a higher level of medical dependency, and hence greater severity of injury with resulting greater neurological impairment and complex rehabilitation needs subsequently. The initial stay on another ward may also be indicative of a delayed neurosurgical presentation either from an unrecognised neurological injury or complication of neurological injury not initially anticipated to require neurosurgical intervention or a neurorehabilitation environment. Another explanation yet might be that this group of patients may be a result of a lack of capacity within the neurosurgical unit and hence patients were having to be accommodated in an environment not specialised for their needs before they arrived at the neurosurgical rehabilitation environment.

In a LOS study focussing on early neurological rehabilitation,^19^ listed diagnoses such as cerebral ischemia, traumatic brain injury, intracerebral haemorrhage and spinal trauma, among others, that frequently necessitate neurological rehabilitation. These diagnoses uniformly displayed an average length of stay exceeding 40 days, signifying the dependence on a high degree of nursing and medical care. This observation parallels the finding in our study of a group of patients with multiple ward transfers leading up to neurosurgical unit admission with greater neurological rehabilitation needs, causing marked shifts in LOS greater than 37 days. Enhanced recognition from trauma policymakers and healthcare providers on the impact on LOS for patients who end up on neurosurgical units following transfers from other inpatient wards could help in addressing the needs of this group of patients and reducing LOS.

There are a number of strengths in this study. This is a huge dataset, which was made accessible through linking the TARN dataset with the SAIL data platform. Through the utilisation of various visualisation techniques, we were able to recognise patterns, trends and relationships within the dataset. We ensured a thorough comprehension of the data, thereby facilitating the identification of key variables and directing subsequent analyses. The boxplot analysis provides a clear visual representation of the central tendency and spread of the scores in the dataset, facilitating a better understanding of the data’s distribution.

A number of limitations exist. Firstly, due to the existence of a certain number of missing values and unlinkable variables for imputation, a certain number of trauma admission records and clinical variables, including time-related data associated with pre-hospital emergencies, surgery code, diagnosis code, complication records, CT scans were excluded from this study.

Variables associated with the documentation of complications have been recognised as key features of length of stay (LOS) in previous studies. For example, number of comorbid conditions,^20^ Charlson Index, Sepsis category and Chronic Disabling Disease score^21^ and complex comorbidities^22^ were found significantly associated with LOS. Secondly, certain social factors, including social status, insurance category, ethnicity and marital status and functional independence, have been examined as variables in LOS modelling in previous studies.^23-25^ These factors were not included in our study. Additional research on their possible association to LOS may be carried out if the data becomes accessible in the future.

Furthermore, while the existing visualisations provide insights into certain variables that could impact LOS, it is essential to validate and understand the relationships between these predictors and LOS through further modelling techniques. Explaining and predicting LOS based on key variables is crucial for scheduling trauma services and allocating healthcare resources effectively. We plan on further research on future datasets to be focussed on the interpretation and prediction of the length of stay with forecasting model comparisons.

## Conclusion

In conclusion, our retrospective study first analysed the trends and patterns of patient length of stay in SWTN, and secondly examining and visualising the association between LOS and various clinical and demographic factors. These findings offer empirical support for allocating medical resources to specialised wards and patients’ rehabilitation. Our findings indicate that elderly patients are vulnerable to extended hospitalisations, which aligns with previous research. Moreover, concerning the types of admission wards, patients admitted to wards who underwent more than 2 ward admissions prior also faced an increased risk of prolonged stay. This observation was particularly dramatic for those transferred to a neurosurgical ward, reflecting the impact on LOS for those with neurorehabilitation needs. In view of these findings, policymakers and healthcare providers should contemplate expanding the allocation of medical resources to this demographic to mitigate the length of stay and optimise associated healthcare costs.

## Supplemental Material

sj-docx-1-rpo-10.1177_27536351241237866 – Supplemental material for Investigating Length of Stay Patterns and Its Predictors in the South Wales Trauma Network Supplemental material, sj-docx-1-rpo-10.1177_27536351241237866 for Investigating Length of Stay Patterns and Its Predictors in the South Wales Trauma Network by Zihao Wang, Bahman Rostami-Tabar, Jane Haider, Mohamed Naim and Javvad Haider in Advances in Rehabilitation Science and Practice
