# Supplementary material for: Investigating Length of Stay Patterns and Its Predictors in the South Wales Trauma Network
Source: Adv Rehabil Sci Pract. 2024 Mar 19;13:27536351241237866. doi: 10.1177/27536351241237866 (PMC10949546; doi:10.1177/27536351241237866)
Supplement: sj-docx-1-rpo-10.1177_27536351241237866 – Supplemental material for Investigating Length of Stay Patterns and Its Predictors in the South Wales Trauma Network [file sj-docx-1-rpo-10.1177_27536351241237866.docx]

## Supplementary tables

**Table 4.** Demographic characteristics comparison within the 2 los groups (p-value is based on the chi-square test)

|  | **Length of stay ≤ 37 days** | **Length of stay > 37 days** | ***p*** |
| --- | --- | --- | --- |
| **Welsh incident** |  |  | < 0.0001 |
| No | 3581 (21.78%) | 192 (12.63%) |  |
| Yes | 12864 (78.22%) | 1328 (87.37%) |  |
| **Welsh resident** |  |  | < 0.0001 |
| No | 1140 (6.93%) | 51 (3.36%) |  |
| Yes | 15305 (93.07%) | 1469 (96.64%) |  |
| **Welsh hospital** |  |  | < 0.0001 |
| No | 3248 (19.75%) | 132 (8.68%) |  |
| Yes | 13197 (80.25%) | 1388 (91.32%) |  |
| **First doctor see patients** |  |  | < 0.05 |
| Associate Specialist | 812 (4.94%) | 61 (4.01%) |  |
| Consultant | 4199 (25.53%) | 390 (25.66%) |  |
| FY / ST 1-2 | 5333 (32.43%) | 498 (32.76%) |  |
| Other | 763 (4.64%) | 69 (4.54%) |  |
| ST 3+ | 4335 (26.36%) | 401 (26.38%) |  |
| ST year unknown | 1003 (6.10%) | 101 (6.64%) |  |
| **Most senior doctor see patients** |  |  | < 0.05 |
| Associate Specialist | 873 (5.31%) | 62 (4.08%) |  |
| Consultant | 6278 (38.18%) | 596 (39.21%) |  |
| FY / ST 1-2 | 3405 (20.71%) | 304 (20.00%) |  |
| Other | 288 (1.75%) | 30 (1.97%) |  |
| ST 3+ | 4554 (27.69%) | 408 (26.84%) |  |
| ST year unknown | 1047 (6.37%) | 120 (7.89%) |  |
